# Demographic Analysis of Patients With Ulcerative Colitis Undergoing Restorative Proctocolectomy With Ileal Pouch-Anal Anastomosis: A Single-Center Retrospective Study

**DOI:** 10.7759/cureus.110905

**Published:** 2026-06-15

**Authors:** Angel Geromin Pérez Queb, Mildred Philippe Ponce, Estefania Contreras Avilés, Valeria Natalie Sebastian Ocampo, Raquel Yazmin López Pérez, Billy Jiménez Bobadilla, Jorge Luis De León Rendón

**Affiliations:** 1 Coloproctology Service, Hospital General de Mexico Dr. Eduardo Liceaga, Mexico City, MEX; 2 Medicine, Universidad Nacional Autónoma de México, Mexico City, MEX; 3 Inflammatory Bowel Disease Clinic, Coloproctology Service, Hospital General de Mexico Dr. Eduardo Liceaga, Mexico City, MEX; 4 Gastrointestinal Endoscopy Service, Hospital General de Mexico Dr. Eduardo Liceaga, Mexico City, MEX

**Keywords:** ileal pouch-anal anastomosis, pouchitis, pouch outcomes, restorative proctocolectomy, ulcerative colitis (uc)

## Abstract

Introduction: Ulcerative colitis (UC) is a chronic relapsing inflammatory bowel disease limited to the colonic mucosa, with rising incidence and prevalence in many regions of the world, including Latin America. A subset of patients requires colectomy for medically refractory disease, acute severe colitis, dysplasia, or malignancy, for whom restorative proctocolectomy with ileal pouch-anal anastomosis (RPC-IPAA) is the preferred restorative procedure. Pouch-related complications -- including pouchitis, chronic antibiotic-refractory pouchitis (CARP), pouch failure, and diagnostic reclassification to Crohn's disease -- represent major clinical challenges; however, published institutional data on RPC-IPAA outcomes from hospital centers in Mexico and Latin America remain exceptionally scarce. This descriptive single-center study aimed to describe the demographic profile, operative characteristics, and predefined pouch-related complications in patients with UC who underwent RPC-IPAA at a tertiary referral center in Mexico.

Methods: A retrospective observational study was conducted at the Inflammatory Bowel Disease Clinic of the Coloproctology Service, Hospital General de México "Dr. Eduardo Liceaga," reviewing medical records of UC patients who underwent RPC-IPAA between 2010 and 2022. Demographic and clinical variables were analyzed using IBM SPSS Statistics, version 29 (IBM Corp., Armonk, NY). Quantitative variables were summarized as mean ± SD and categorical variables as frequencies and percentages. Pouchitis was diagnosed using the Pouchitis Disease Activity Index (PDAI ≥7). CARP was defined as active pouchitis (PDAI ≥7) persisting despite at least four consecutive weeks of antibiotic therapy and failure of at least two sequential antibiotic regimens. Postoperative follow-up was calculated from the date of pouch creation to the last clinical encounter.

Results: Fourteen patients were included; 8 (57.1%) were women. Mean age was 34.86 ± 7.94 years. All patients had Montreal E3 pancolitis. The mean interval between UC diagnosis and pouch creation was 3.86 ± 1.81 years. The median postoperative follow-up was 6.2 years (IQR: 3.4-9.1 years; range: 0.5-12.0 years). J-pouch construction was performed in 13 (92.9%) patients and D-pouch in 1 (7.1%). One (7.1%) patient experienced pouch failure and underwent W-pouch reconstruction. One (7.1%) patient originally diagnosed with UC E3 was subsequently reclassified as Crohn's disease (Montreal A2L4B2) and is currently receiving anti-interleukin-12/23 therapy (ustekinumab). Pouchitis occurred in 4 (28.6%) patients; all developed CARP and are currently being treated with anti-integrin therapy (vedolizumab).

Conclusions: In this descriptive single-center study, RPC-IPAA was predominantly performed with J-pouch construction, achieving durable pouch preservation over a median follow-up of 6.2 years. Pouch-related complications -- including CARP, pouch failure, and diagnostic reclassification to Crohn's disease -- emerged as important sources of morbidity requiring escalation to advanced biologic therapy and, in selected cases, surgical reintervention. These findings underscore that successful pouch surgery depends on structured individualized surveillance, dedicated multidisciplinary care, and timely access to biologic therapies throughout the postoperative course.

## Introduction

Ulcerative colitis (UC) is a chronic inflammatory bowel disease restricted to the colonic mucosa and rectum. Its prevalence is highest in North America and Europe, but the incidence of UC has been rising in Latin America and other newly industrialized regions, reflecting a growing global burden of disease [1]. Although medical therapy has evolved substantially, colectomy remains necessary in a subset of patients because of medically refractory disease, acute severe colitis, dysplasia, or colorectal neoplasia [2,3].

For patients requiring colectomy, restorative proctocolectomy with ileal pouch-anal anastomosis (RPC-IPAA) remains the preferred restorative procedure because it removes the diseased colon and rectum while preserving intestinal continuity and transanal defecation [2,3]. Over time, the J-pouch has become the dominant reconstructive design owing to its technical feasibility and favorable functional outcomes, while refinements in operative technique have continued to optimize pouch creation and anastomotic safety [4-7].

Despite these advantages, morbidity after RPC-IPAA remains substantial over time. Pouchitis is the most frequent complication, but the clinical spectrum also includes chronic antibiotic-refractory pouchitis (CARP), pouch failure, and postoperative diagnostic reclassification to Crohn's disease, all of which may significantly affect quality of life and sustained pouch survival [8-16]. These complications increasingly require advanced biologic therapy and prolonged multidisciplinary surveillance [10-16].

The present study aimed to describe the demographic profile, operative characteristics, and predefined pouch-related complications -- specifically pouchitis, CARP, pouch failure, and postoperative diagnostic reclassification to Crohn's disease -- in patients with UC who underwent RPC-IPAA at a tertiary referral center in Mexico. Of note, published institutional data on RPC-IPAA outcomes from hospital centers in Mexico and Latin America remain exceptionally scarce, underscoring the need for regional descriptive studies to inform patient counseling and pouch program development.

This study was previously presented at Advances in Inflammatory Bowel Diseases (AIBD) 2024, Orlando, Florida, USA, December 9-11, 2024; the 66th Annual Meeting of the Society for Surgery of the Alimentary Tract (SSAT), San Diego, California, USA, May 3-6, 2025; and the American Society of Colon and Rectal Surgeons (ASCRS) Annual Scientific Meeting, San Diego, California, USA, May 10-13, 2025. Preliminary findings were published in abstract form.

## Materials and methods

A retrospective observational cohort study was conducted at the Inflammatory Bowel Disease Clinic of the Coloproctology Service, Hospital General de México "Dr. Eduardo Liceaga," a tertiary referral center in Mexico City, Mexico. Medical records from January 2010 to December 2022 were reviewed to identify all consecutive patients with an established diagnosis of UC who underwent RPC-IPAA.

Adult patients with a confirmed diagnosis of UC based on clinical, endoscopic, radiologic, and histopathologic findings were eligible for inclusion. Only patients who underwent RPC-IPAA during the study period and had complete clinical records with available postoperative follow-up information were included in the analysis. Patients with a preoperative diagnosis of Crohn's disease or indeterminate colitis were excluded, as were those who underwent colectomy without ileal pouch reconstruction. Patients with incomplete medical records or insufficient follow-up data were also excluded. The primary indication for surgery was medically refractory disease, defined as inadequate response, loss of response, intolerance, or dependence on conventional and/or advanced medical therapies, as determined by the treating multidisciplinary team.

Clinical data were extracted from institutional electronic and paper medical records using a pre-specified standardized data collection form applied uniformly to all records. Data extraction was performed independently by two investigators; discrepancies were resolved by consensus review. Demographic variables included sex and age at the time of surgery. Clinical variables comprised disease extent according to the Montreal Classification for UC -- E1 (ulcerative proctitis), E2 (left-sided colitis), and E3 (extensive colitis extending proximal to the splenic flexure) [17] -- the interval between UC diagnosis and pouch creation, pouch configuration, and the following pouch-related outcomes: occurrence of pouchitis, progression to CARP, pouch failure, postoperative biologic therapy, and diagnostic reclassification to Crohn's disease during follow-up. Pouchitis was diagnosed using the Pouchitis Disease Activity Index (PDAI), with a composite score ≥7 required to establish the diagnosis [8]. The PDAI integrates three subscales: a clinical subscale (stool frequency above individual baseline, rectal bleeding, fecal urgency or abdominal cramping, and fever >37.8°C); an endoscopic subscale (edema, granularity, friability, loss of vascular pattern, mucous exudate, and ulceration); and a histological subscale (acute polymorphonuclear leukocyte infiltration and ulceration density per high-power field). CARP was defined as active pouchitis (PDAI ≥7) persisting despite at least four consecutive weeks of antibiotic therapy -- ciprofloxacin (500 mg twice daily) and/or metronidazole (400 mg three times daily) -- and failure of at least two sequential antibiotic regimens [10-12]. Postoperative diagnostic reclassification to Crohn's disease required multidisciplinary team consensus upon identification of histological and/or radiological features irreconcilable with UC, including granulomatous inflammation on biopsy, transmural disease, proximal small bowel involvement, or perianal disease [15,17]. Single-modality findings were not sufficient, and multidisciplinary agreement was mandatory in all cases. Postoperative follow-up duration was calculated from the date of pouch creation to the date of the last clinical encounter available in the institutional medical record within the study period.

The primary outcome of interest was the occurrence of pouch-related complications during follow-up, comprising four predefined endpoints: (i) pouchitis, diagnosed by PDAI ≥7 [8]; (ii) progression to CARP as defined above [10-12]; (iii) pouch failure requiring excision or permanent diversion [13]; and (iv) postoperative diagnostic reclassification to Crohn's disease [15]. Secondary descriptive objectives included characterization of the demographic profile, Montreal disease extent at surgery [17], surgical technique employed, and postoperative biologic therapy requirements.

Statistical analyses were performed using IBM SPSS Statistics, version 29 (IBM Corp., Armonk, NY). Continuous variables are presented as mean ± SD, whereas categorical variables are reported as absolute frequencies and percentages. Given the descriptive nature of the study and the limited sample size, no comparative or inferential statistical analyses were performed.

This study forms part of the institutional research activities of the Inflammatory Bowel Disease Clinic of the Coloproctology Service, Hospital General de México "Dr. Eduardo Liceaga." The study protocol and request for waiver of informed consent were reviewed and approved by the Research Ethics Committee of Hospital General de México "Dr. Eduardo Liceaga." Due to the retrospective design, the exclusive use of previously collected clinical data, the absence of direct patient contact, and the minimal risk posed to participants, the requirement for informed consent was waived by the Ethics Committee. All patient information was anonymized before analysis and handled in accordance with institutional regulations and the ethical principles outlined in the Declaration of Helsinki.

## Results

Fourteen patients with UC who underwent RPC-IPAA between 2010 and 2022 were included in the analysis. Of these, 8 (57.1%) patients were women, and 6 (42.9%) were men. The mean age at the time of surgery was 34.86 ± 7.94 years. According to the Montreal classification, all patients presented with extensive disease (E3 pancolitis), reflecting the severity and extent of colonic involvement within this surgical cohort. The mean interval between the initial diagnosis of UC and pouch creation was 3.86 ± 1.81 years. The median postoperative follow-up was 6.2 years (IQR: 3.4-9.1 years; range: 0.5-12.0 years). 

With respect to reconstructive techniques, total proctocolectomy followed by J-pouch ileal reservoir construction was the predominant surgical approach, performed in 13 (92.9%) patients. Only 1 (7.1%) patient underwent D-pouch construction. During the follow-up period, pouch failure occurred in 1 (7.1%) patient, necessitating pouch excision and subsequent reconstruction with a W-pouch configuration.

Notably, 1 (7.1%) patient, initially diagnosed with UC classified as Montreal E3, underwent diagnostic reclassification to Crohn’s disease during follow-up. This patient is receiving anti-interleukin-12/23 therapy (ustekinumab) with ongoing clinical surveillance.

Pouch-related inflammatory complications were observed in 4 (28.6%) patients. Importantly, all affected individuals progressed to CARP, representing a clinically significant phenotype associated with substantial morbidity and therapeutic complexity. These patients required escalation to advanced medical therapy and are currently being treated with the anti-integrin agent vedolizumab.

**Figure 1:**
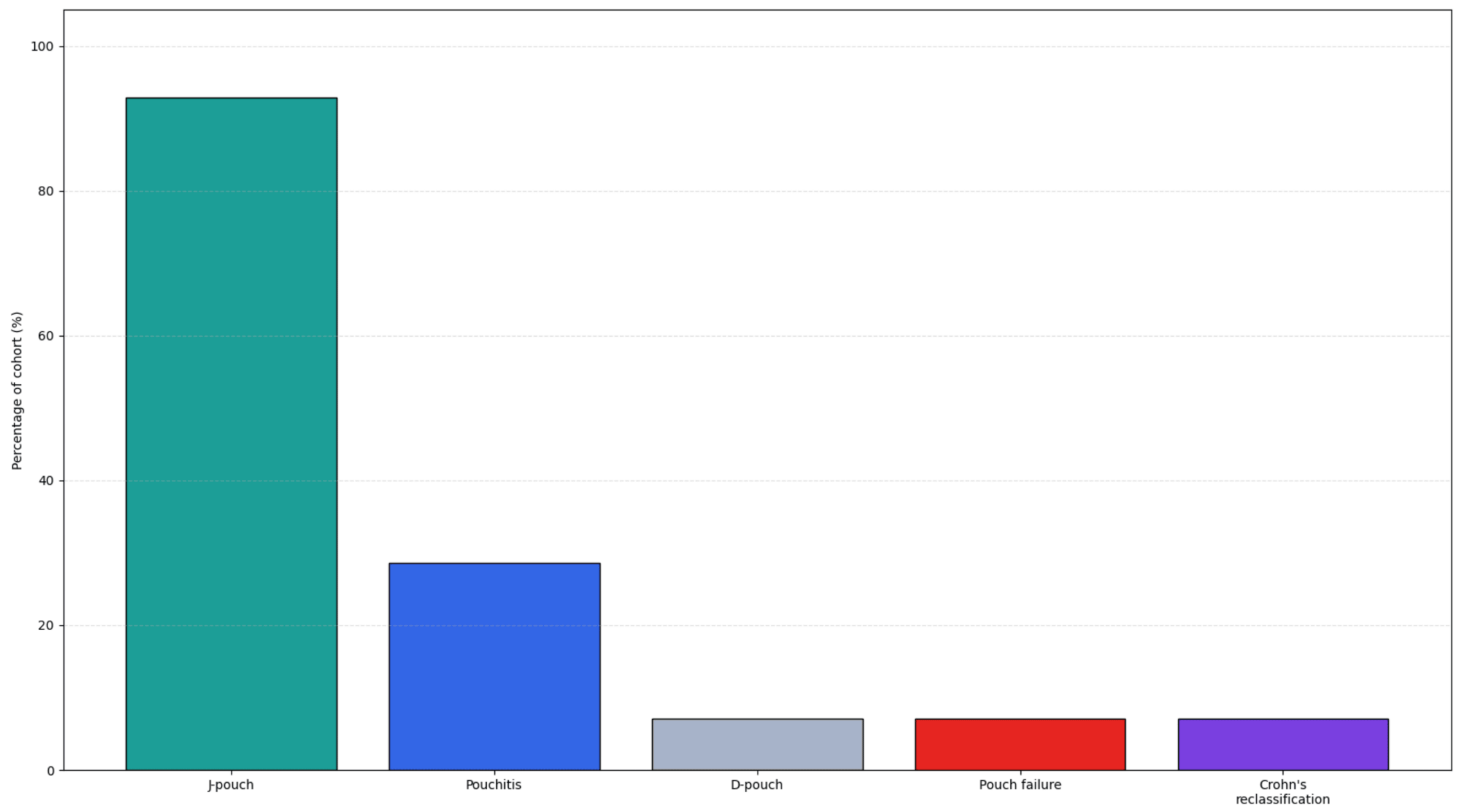
Cohort overview: pouch configuration and outcomes

Overall, despite durable pouch preservation in the majority of patients over a median follow-up of 6.2 years, the occurrence of chronic pouch inflammation, pouch failure, and phenotypic conversion to Crohn's disease underscores the need for structured individualized surveillance and multidisciplinary management strategies after RPC-IPAA. The demographic, clinical, and surgical characteristics of the cohort are summarized in Table 1 and Figure 1.

**Table 1:** Demographic and clinical characteristics of the study cohort (n=14) IL, interleukin; SD, standard deviation; UC, ulcerative colitis.

Variable	Value
Female sex, n (%)	8 (57.1%)
Mean age at surgery, years (mean ± SD)	34.86 ± 7.94
UC disease extent: Montreal E3 pancolitis, n (%)	14 (100%)
Interval UC diagnosis to pouch creation, years (mean ± SD)	3.86 ± 1.81
J-pouch construction, n (%)	13 (92.9%)
D-pouch construction, n (%)	1 (7.1%)
Pouch failure, n (%)	1 (7.1%)
Pouchitis (chronic antibiotic-refractory), n (%)	4 (28.6%)
Diagnostic reclassification to Crohn's disease (Montreal A2L4B2), n (%)	1 (7.1%)
Biologic therapy after pouch construction	
Vedolizumab (anti-integrin), n (%)	4 (28.6%)
Ustekinumab (anti-IL-12/23), n (%)	1 (7.1%)

## Discussion

This retrospective single-center study provides a descriptive characterization of the demographic profile, surgical features, and pouch-related outcomes of patients with UC who underwent RPC-IPAA at a tertiary referral center in Mexico. The principal findings include a predominance of J-pouch reconstruction, a substantial burden of chronic refractory pouchitis requiring biologic therapy, a low rate of pouch failure with successful salvage in most cases, and a small proportion of patients subsequently reclassified as having Crohn’s disease, highlighting the phenotypic complexity and therapeutic challenges of this population.

The predominance of J-pouch construction (92.9%) in this series aligns with established international practice. Large-volume series have consistently confirmed the J-pouch as the most reproducible and widely adopted configuration, combining technical simplicity with acceptable functional outcomes [4,5]. Comparative studies have not demonstrated a consistent functional superiority of more complex designs such as the S- or W-pouch, reinforcing the J-pouch as the default choice in routine practice [5]. In our series, one patient underwent D-pouch construction, a modification designed to optimize reservoir reach in anatomically challenging cases -- an infrequent but recognized alternative in specialized settings [18].

Pouchitis was observed in 28.6% of the cohort, a proportion consistent with published incidence rates across varying follow-up periods. Review data indicate that acute pouchitis affects up to 20% of patients within the first year after ileal pouch-anal anastomosis (IPAA) and may cumulatively affect 70% or more in series with extended follow-up [8]. Critically, all four affected patients in our cohort evolved to CARP, a phenotype associated with substantially greater morbidity and quality-of-life impairment than episodic disease [9,10]. CARP affects approximately 10%-15% of patients who develop pouchitis and represents the most therapeutically challenging pouch inflammatory phenotype [10].

The therapeutic profile of our CARP subgroup is particularly relevant: all four affected patients are currently receiving vedolizumab, a gut-selective anti-integrin biologic. This aligns with the contemporary shift in chronic refractory pouch inflammation management toward advanced targeted therapy. Systematic review evidence supports the use of biologics in this setting, with anti-tumor necrosis factor agents, vedolizumab, and other advanced therapies showing benefit in selected patients [11]. More importantly, the EARNEST trial provided randomized controlled evidence that vedolizumab was superior to placebo for inducing remission in chronic pouchitis after IPAA, supporting its use as a preferred first-line advanced biologic option when antibiotic-refractory disease persists [12]. The exclusive use of vedolizumab in our series is therefore concordant with an evidence-based, gut-selective strategy for CARP management in contemporary inflammatory bowel disease (IBD) practice.

Pouch failure occurred in one patient (7.1%), who underwent successful W-pouch reconstruction after pouch excision. Despite being numerically infrequent, pouch failure carries substantial functional, psychological, and surgical burden. Meta-analytic data report overall prevalence rates ranging from 2% to 15% depending on cohort characteristics and follow-up duration [13]. In the biologic era, prior biologic exposure before colectomy and Crohn-like pouch inflammation are increasingly recognized as independent risk factors for pouch loss [14], highlighting the importance of thorough preoperative phenotypic characterization in the multidisciplinary evaluation of surgical candidates.

Perhaps the most clinically instructive finding in our series is the later reclassification of one patient from UC E3 to Crohn's disease during follow-up. This patient is currently receiving ustekinumab, an anti-interleukin-12/23 antibody. This case illustrates several important clinical points. First, diagnostic reclassification after IPAA is a well-documented phenomenon, with pooled estimates from systematic reviews placing the risk at 5%-16% of patients operated for apparent UC [15]. Second, reclassification to Crohn's disease substantially increases the risk of pouch failure and inflammatory complications, frequently requiring advanced biologic therapy [16]. Third, active phenotypic surveillance must be sustained beyond the initial postoperative period and maintained throughout the entire postoperative course [16,19].

The use of ustekinumab in the patient, subsequently reclassified as having Crohn’s disease, was justified by the well-established efficacy of anti-IL-12/23 therapy in Crohn’s disease. This finding underscores the importance of an individualized therapeutic strategy for Crohn’s disease of the pouch, tailored to the underlying phenotypic characteristics of each patient.

Pouch survival and functional outcomes over extended follow-up periods provide the broader context for interpreting individual complication events. Single-center series from high-volume centers have reported pouch survival rates exceeding 90% at 20 years, with stable functional performance despite aging [19-21]. Quality-of-life assessments using the CGQL instrument have confirmed satisfactory outcomes in the majority of patients, suggesting that IPAA remains an appropriate restorative strategy despite the inflammatory complications observed in a subset of patients [19-22].

Our study also adds to the limited but growing body of institutional evidence from Mexico. National consensus recommendations position RPC-IPAA as the preferred restorative surgery for UC in Mexico, and prior institutional series have demonstrated technical feasibility [23-25]. The present cohort extends this experience by documenting a detailed postoperative complication profile - including CARP treated with vedolizumab and Crohn's disease reclassification treated with ustekinumab - that has not been previously reported from a Mexican IBD center.

This study has several limitations that must be acknowledged. First, the small sample size (n=14) limits statistical power and precludes inferential or multivariable analyses; all reported rates represent observed proportions within this cohort and should not be extrapolated as population-level estimates. Second, the retrospective design restricts analysis to variables available in existing medical records, precluding systematic collection of functional pouch outcomes, validated quality-of-life measures, PDAI subscores at each clinical encounter, preoperative biologic exposure -- increasingly recognized as an independent predictor of pouch failure -- and exact dates of complication onset. Third, as a tertiary referral center, the institution receives a disproportionately complex and refractory case mix, which likely enriches complication rates relative to community surgical settings and limits generalizability. Fourth, the retrospective diagnosis of Crohn's disease reclassification carries inherent uncertainty, as histopathological reassessment was not performed at a single standardized time point. Fifth, the heterogeneous follow-up duration across patients (range: 0.5-12.0 years) limits comparability of outcomes within the cohort. Sixth, the absence of a comparator group precludes any comparative inference. Despite these constraints, this series represents one of the few detailed institutional reports of RPC-IPAA outcomes from Mexico, contributing data of relevance to pouch program development and patient counseling in Latin America [14].

## Conclusions

This descriptive single-center study represents one of the few institutional reports of RPC-IPAA for UC from Mexico and Latin America, where published evidence from hospital centers on pouch outcomes remains exceptionally scarce. J-pouch reconstruction achieved durable pouch preservation over a median follow-up of 6.2 years; nevertheless, clinically significant pouch-related complications -- including CARP, pouch failure, and diagnostic reclassification to Crohn's disease -- emerged as important sources of morbidity requiring escalation to advanced biologic therapy and, in selected cases, surgical reintervention. These findings underscore that successful pouch surgery extends well beyond the operative procedure itself and depends on structured individualized surveillance, dedicated multidisciplinary care, and timely access to biologic therapies throughout the postoperative course.
